# The Complete Genome of a Novel Typical Species *Thiocapsa bogorovii* and Analysis of Its Central Metabolic Pathways

**DOI:** 10.3390/microorganisms12020391

**Published:** 2024-02-15

**Authors:** Ekaterina Petushkova, Makhmadyusuf Khasimov, Ekaterina Mayorova, Yanina Delegan, Ekaterina Frantsuzova, Alexander Bogun, Elena Galkina, Anatoly Tsygankov

**Affiliations:** 1 Institute of Basic Biological Problems, Federal Research Center “Pushchino Scientific Center for Biological Research of Russian Academy of Sciences” (FRC PSCBR RAS), 142290 Pushchino, Moscow Region, Russia; hasimov94@mail.ru (M.K.); ekaterina.majorova.97@mail.ru (E.M.); ttt-00@mail.ru (A.T.); 2 Institute of Biochemistry and Physiology of Microorganisms, Federal Research Center “Pushchino Scientific Center for Biological Research of Russian Academy of Sciences” (FRC PSCBR RAS), 142290 Pushchino, Moscow Region, Russia; mewgia@yandex.ru (Y.D.); frantsuzova.ee@gmail.com (E.F.); bogun62@mail.ru (A.B.); 3 State Research Center for Applied Microbiology and Biotechnology, 142279 Obolensk, Moscow Region, Russia; elen12123@yandex.ru

**Keywords:** *Thiocapsa roseopersicina* BBS, *Thiocapsa bogorovii* BBS, purple sulfur bacterium, genome sequencing, reclassification

## Abstract

The purple sulfur bacterium *Thiocapsa roseopersicina* BBS is interesting from both fundamental and practical points of view. It possesses a thermostable HydSL hydrogenase, which is involved in the reaction of reversible hydrogen activation and a unique reaction of sulfur reduction to hydrogen sulfide. It is a very promising enzyme for enzymatic hydrogenase electrodes. There are speculations that HydSL hydrogenase of purple bacteria is closely related to sulfur metabolism, but confirmation is required. For that, the full genome sequence is necessary. Here, we sequenced and assembled the complete genome of this bacterium. The analysis of the obtained whole genome, through an integrative approach that comprised estimating the Average Nucleotide Identity (ANI) and digital DNA-DNA hybridization (DDH) parameters, allowed for validation of the systematic position of *T. roseopersicina* as *T. bogorovii* BBS. For the first time, we have assembled the whole genome of this typical strain of a new bacterial species and carried out its functional description against another purple sulfur bacterium: *Allochromatium vinosum* DSM 180T. We refined the automatic annotation of the whole genome of the bacteria *T. bogorovii* BBS and localized the genomic positions of several studied genes, including those involved in sulfur metabolism and genes encoding the enzymes required for the TCA and glyoxylate cycles and other central metabolic pathways. Eleven additional genes coding proteins involved in pigment biosynthesis was found.

## 1. Introduction

The purple sulfur bacterium *T. roseopersicina* BBS belongs to the *Chromatiaceae* family from the class *Gammaproteobacteria*. This strain was isolated from the White Sea Estuary in 1969 [1]. Bacterial cells are spherical with a diameter of 1.0–1.5 microns. The color of the culture depends on the growth conditions and the composition of growth media and may range from pinkish red to light pink in cases of accumulating molecular sulfur. The bacteria are Gram negative. Despite the absence of flagella, the bacteria are capable of spontaneous impulse movements in wet microscopic samples. The culture grows in the pH range of 6.0–8.0 (optimum pH is 7.0–7.5) and in the presence of NaCl (up to 5%, the optimum content being 1–2%). *T. roseopersicina* BBS is a B_12_ auxotroph. It reproduces by a binary fission. The culture accumulates polyphosphate granules, poly-β-butyrate, and polysaccharides as storage compounds. It can grow under chemolithotrophic conditions [2]. Over the past years, the metabolism of hydrogen, sulfur, and carbon as well as the biosynthesis of the photosynthetic pigments in these bacteria have been extensively studied at both biochemical and physiological levels [3,4,5,6,7,8]. Special attention has been paid to the hydrogenases of this species, their structure, maturation, and cellular functions as well as the genes encoding the hydrogenases and the enzymes responsible for their synthesis [9,10,11,12,13,14]. However, for future research and practical application of this bacterium, the full genome sequence is important.

The aim of this work was to assemble the data of the whole genome of *Thiocapsa* sp. BBS; give a short description; analyze the genetic potential of this bacterium in sulfur metabolism, comparing it with the most studied purple sulfur bacterium, *Allochromatium vinosum*, for research into HydSL hydrogenase’s role in sulfur metabolism; present the genetic reason for the autotrophic growth of the bacterium; check for the presence of the citric acid cycle; and validate systematic position of the studied bacterium.

## 2. Materials and Methods

### 2.1. Growth Conditions

*T. roseopersicina* BBS was obtained from the culture collection of the Department of Microbiology, Moscow State University. The bacterium was cultivated at 28 °C in a modified Pfennig medium [1] containing NaHCO_3_ (0.2%), Na_2_S 9H_2_O (0.1%), sodium thiosulfate (0.2%), B_12_ (20 µg/ml), and NaCl (2%). Cells were cultivated under phototrophic conditions at an illumination intensity of 60 W/m^2^ in 250 ml flasks with stoppers.

### 2.2. Genome Sequencing, Assembly, and Annotation

Genomic DNA was isolated from the biomass of a fresh culture (a colony) of *T. roseopersicina* BBS grown under anaerobic photoheterotrophic conditions on modified Pfennig’s medium in the presence of 0.2% sodium acetate using the Monarch^®^ HMW DNA Extraction Kit (T3050L, NEB). Sequencing was performed using the MinION sequencer with an R9.4.1 flow cell (Oxford Nanopore Technologies [ONT]) in the facilities of the State Research Center for Applied Microbiology and Biotechnology (SRCAMB, Obolensk, Russia). The library was prepared using the Rapid Barcoding Kit (cat. # SQK-RBK00). Guppy version 3.2.4 software was used for base calling, which yielded a total of 338.5 Mb distributed in 179,494 reads. Reads were filtered based on the quality metric (Q > 10).

The same DNA sample was sequenced using the Illumina NovaSeq 6000 platform with an S2 reagent kit (catalog number 20012861; 2 × 100 bp; BioSpark, Moscow, Russia). A paired-end library was prepared with the Kapa HyperPlus kit (Roche, Basel, Switzerland).

Quality control of Illumina reads was carried out using FastQC. (http://www.bioinformatics.babraham.ac.uk/projects/fastqc accessed on 1 June 2023). Illumina and Nanopore reads were used for hybrid assembly with SPAdes version 3.15.2 [15]. Nanopore reads were assembled into contigs using the Flye assembler version 2.6 [16]. SPAdes contigs were then combined into replicons using Flye data as a reference. Illumina reads were used to correct Nanopore errors using Bowtie2 version 2.3.5.1 [17] and Pilon version 1.23 [18] software. Default settings were used for all software. Circularization of the ends of a chromosome was confirmed by overlapping ends as well as by visualization in the Tablet program [19].

Data were submitted to the GenBank database under the following accession numbers: BioProject—PRJNA224116, BioSample—SAMN23799607, and GenBank—CP089309.1

The assembled genome was annotated using Prokka [20] and RAST [21]. The functions of some proteins were checked manually using BLAST. The phylogenetic tree was constructed by the neighbor-joining method using the REALPHY service [22]. Genome sequences of *Thiocapsa* strains required for constructing the phylogenetic tree were taken from the WGS database (https://www.ncbi.nlm.nih.gov/Traces/wgs/?view=wgs, accessed on 11 October 2021). The circular map was created using DNAPlotter [23].

The ANI value was calculated using the EzBioCloud service [24]. The DDH parameter was calculated using the Genome-to-Genome Distance Calculator 2.1 service [25]. We used the Kyoto Encyclopedia of Genes and Genomes (KEGG) [26] to perform functional annotation using the blastp module.

Horizontal gene transfer regions (HGT regions) were searched using Alien Hunter [27]. CRISPR/Cas elements were searched using CRISPR/Cas Finder [28].

Metabolic pathways at the level of enzymes and their genes in *T. roseopersicina* BBS were analyzed using information from the KEGG Pathway database for prokaryotes, as well as records for substrates and products of biochemical reactions in KEGG Compounds. Searching for homologous genes in the *T. roseopersicina* BBS genome was performed using tBLASTn (by amino acid sequences of six open reading frames of the genome [29]). The best-characterized gene of a prokaryotic organism (i.e., having an experimentally proven function of interest) was chosen as a query for analysis. If highly homologous genes were absent, the test was repeated with less-characterized sequences (from purple bacteria only) as queries. The algorithm parameters were set to default values. The criteria for finding the gene of interest were as follows: for protein sequences, identity > 40% and alignment length > 100 Aa, Bit Score > 50, and e-value (the expectation value) ≤ 10^−6^ indicated the presence of the gene (provided that all other conditions were met); whereas e-value > 10^−6^ implied that the gene was absent. If this did not reveal the putative gene, the gene was considered as absent. We detected a small number of genes satisfying the search criteria. Amino acid sequences of the proteins from the UniProt database were used as search templates [30].

## 3. Results and Discussion

### 3.1. General Characteristics of the T. roseopersicina BBS Genome

**Figure 1:**
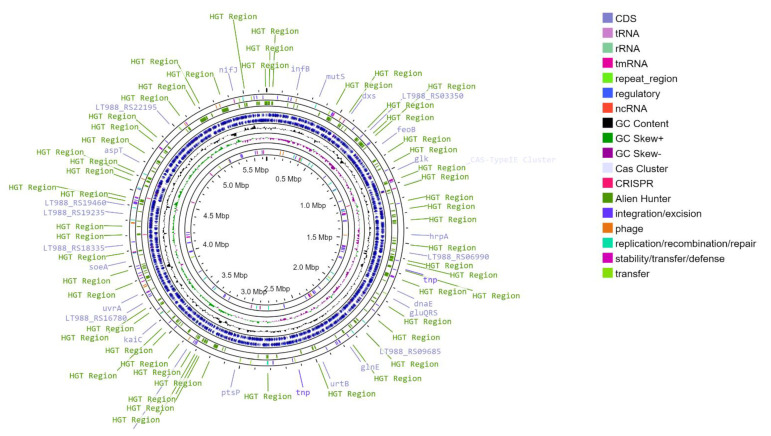
Circular genomic map of *T. roseopersicina* BBS chromosome.

**Figure 2:**
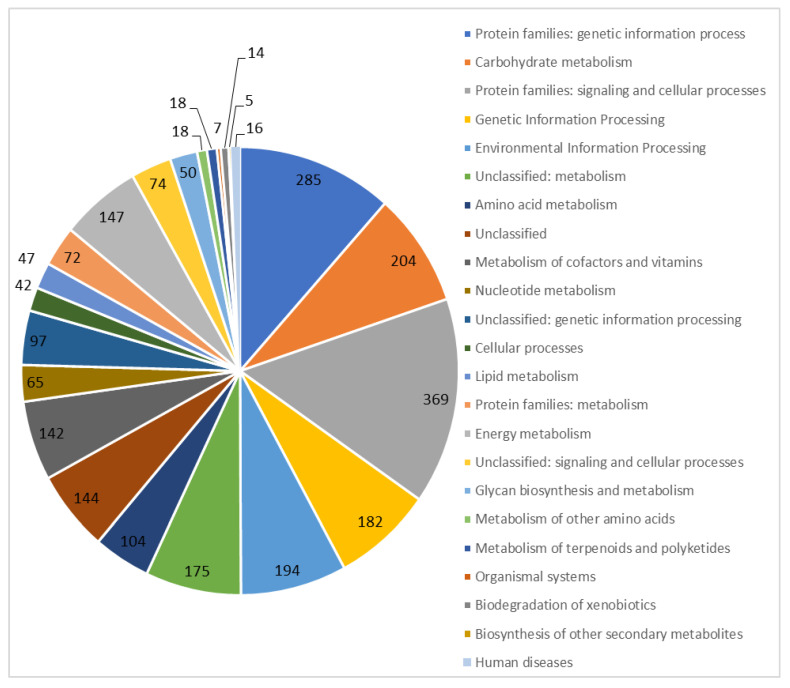
Functional annotation of the genomic DNA of *T. roseopersicina* BBS.

We sequenced and assembled the genome of *T. roseopersicina* BBS. Its main statistical features are shown in Table 1. The *T. roseopersicina* BBS DNA consists of a circular chromosome 5,649,927 bp long (with a GC content of 63.94%), and plasmids are absent (Figure 1). The chromosome contains 5036 genes, 2 rRNA clusters (5S, 16S, 23S), and 51 tRNAs. Of 4978 protein-coding sequences, 2468 (49.6%) were functionally annotated (Figure 2).

### 3.2. Validating the Taxonomic Position of the BBS Strain

The genus *Thiocapsa* currently comprises nine species. Two of them have not been validated at the time of preparing this manuscript (LPSN - List of Prokaryotic names with Standing in Nomenclature, https://www.bacterio.net/, accessed on 1 June 2023). One of the two non-validated species includes the strain under consideration: the reclassification of the *T. roseopersicina* BBS species into a novel species *T. bogorovii* BBS was suggested by Tourova et al. in 2009 with BBS as a type (and single at the moment) strain [31]. However, these data require further validation using a full genome, since GenBank contains only fragmented data on small sequences of *T. roseopersicina* BBS genomic DNA up to 40,000 base pairs in size (AF528191.1, JF712872.1, etc.). Therefore, it has not previously been possible to determine the extent to which this bacterium differs from the typical T*. roseopersicina* strain. 

Analyzing the obtained whole genome of *T. roseopersicina* BBS through an integrated approach that included calculating the ANI and DDH parameters resulted in validation of the systematic position of *T. bogorovii* BBS. This bacterium is not closely related to any typical strain of the species from the *Thiocapsa* genus (*T. marina*, *T. rosea*, *T. imhoffii*, and *T. roseopersicina*) (Table 2). Thus, for the first time, we obtained the assembled whole genome of *T. bogorovii BBS* and confirmed the relevance of its classification into a novel typical species of the *Thiocapsa* genus. 

The systematic position of *T. bogorovii* BBS explains the existence of substantial differences in bacterial physiology from *T. roseopersicina* DSM 217T. In particular, *T. bogorovii* BBS is characterized by the absence of assimilatory sulfate reduction, vitamin B12 auxotrophy, and higher optimal values of growth medium salinity as well as the distinct range of utilized organic compounds [1].

### 3.3. A Comparison of the Whole Genomes of T. bogorovii BBS and Alc. Vinosum DSM 180^T^

To study the specificities of sulfur metabolism and the role of the involved hydrogenases in *T. bogorovii* BBS, the whole sequence of its genome was characterized and compared with the genome of another representative of the *Chromatiaceae* family, *Alc. vinosum* DSM 180 ^T^, since its sulfur metabolism has been studied in detail. The whole sequence of Alc. vinosum DSM 180^T^ consists of one circular chromosome and two plasmids [32]. Similar to *T. bogorovii* BBS, this bacterium is capable of phototrophic growth (anaerobic in the light) and chemotrophic growth (aerobic in the dark). Data on the major features of the genetic categories are given in Table 3. So, these bacteria belong to distinct families of purple sulfur bacteria and are very different in genome composition. Nevertheless, sulfur metabolism is the common features between them and it allows us to compare them in this respect.

#### 3.3.1. Pigment Biosynthesis and Photocomplexes

The photosynthetic apparatus of many purple bacteria consists of three types of pigment-protein complexes: two light-harvesting antenna complexes (LHI and LHII) capture photons of different wavelengths and transfer the excitation energy to the reaction center (RC, complex III) [33]. The core antenna LH1 is adjacent to the RC, forming an open ring [34]. The LH2 peripheral antenna is located at the periphery. There are three structural types of RC–LH1 complexes in purple bacteria [35]. The gaps in the LH1 ring are considered to be necessary for the penetration of the ubiquinone that links RC with the cytochrome *bc_1_* complex. In *Cereibacter sphaeroides*, the PufX protein is present in each monomer of the dimeric RC–LH1 [36]. In the purple non-sulfur bacterium *Rhodopseudomonas palustris* [37], RC exists in the form of the RC–LH1 monomer and contains the protein W (similar to PufX). The third structural type of RC–LH1 complex has been identified in the purple sulfur bacterium *Thermochromatium tepidum* [38]. In this bacterium, RC exists as the RC–LH1 monomer and does not comprise proteins similar to the PufX or W proteins (however, the LH1 ring is open containing channels). Recently, a fourth form of RC–LH1 with a closed ring has been reported in purple bacteria [39]. 

Gene clusters in *T. bogorovii* BBS related to photosynthesis have been investigated earlier. The localization of several genes is uncommon compared to other photosynthetic bacteria [8]. The obtained whole genome of these bacteria allowed for specification of the localization of the enzymes that belonged to the specific pathway of carotenoid synthesis in *T. bogorovii* BBS, the main carotenoid being spirilloxanthin [1,8]. It also allowed for identification of the genes of the light-harvesting photosynthetic complexes and the genes related to the synthesis of bacteriochlorophyll *a*, which are located outside of the previously studied genomic fragments (Table 4). Sequencing errors (substitutions and deletions) were detected in some previously characterized regions. According to the genomic analysis performed, the genes encoding the proteins W and PufX are absent from both *T. bogorovii* BBS and *Alc. vinosum* DSM 180^T^.

#### 3.3.2. Autotrophy and RuBisCO

In purple bacteria, the major pathway for CO_2_ assimilation under autotrophic conditions is the reductive pentose phosphate cycle (Calvin–Benson–Bassham cycle) [40]. Ribulose-1,5-bisphosphate carboxylase/oxygenase (RuBisCO), Phosphoribulokinase (PRK), and Sedoheptulose-bisphosphatase (SBP) are the enzymes that function exclusively in this cycle [41]. Together, RuBisCo and PRK can indicate that this cycle is present in the organism in question. Enzymes that differ from the typical plant form of RuBisCo have been identified in various microorganisms. There are four main forms of RuBisCO [42], with RuBisCO forms I, II, and III exhibiting carboxylase and oxygenase activity, but under potentially different physiological conditions. Form III has been found only in archaea and has therefore been allocated into a separate category. Form IV includes the RubisCO-like protein (RLP). This enzyme does not catalyze ribulose 1,5-bisphosphate-dependent CO_2_ fixation; however, it might be involved in sulfur metabolism and stress responses [43,44] as well as in the methionine salvage pathway [45]. The function of RLP is unknown in many organisms. RuBisCO activity has been detected in the *T. roseopersicina* DSM 217T and *T. bogorovii* BBS cultures [3,7,46]. The peculiarity of the BBS strain is the insensitivity of RuBisCO synthesis to molecular oxygen [3]. In contrast, RuBisCO synthesis is suppressed by oxygen in most purple bacteria [40].

The green-like form I RuBisCO was the only one obtained and sequenced using specific oligonucleotide primers in *T. roseopersicina* DSM 217T and *T. bogorovii* BBS [31], whereas *Alc. vinosum* DSM 180^T^ was shown to contain two forms (the green-like form I RuBisCO and the red-like form II RuBisCO).

Analysis of the *T. bogorovii* BBS genome using the RAST 2 algorithm revealed two copies of the gene encoding the green-like RuBisCO form I. It is worth noting that the genes encoding the small subunits are different, and their nucleotide sequences have 70% identity (using standard settings of the Nucleotide BLAST algorithm). Using tBlastn, the bacterial genome was shown to contain a gene (LT988_08840, 1944337 to 1945626 base pairs) encoding a protein product with 71% identity of amino acid sequence of the form IV “RuBisCO-like” protein of *Alc. vinosum* DSM 180^T^. The role of RLP in *T. bogorovii* BBS has not yet been elucidated.

The structural genes encoding two enzymes of the RuBisCO form I are far apart, and their genetic neighborhoods are different, similar to the RuBisCO genes in *Alc. vinosum* DSM 180^T^ [32]. In one case, the RuBisCO structural genes (LT988_02750, rubisco L 566332–567750 bp and LT988_02755, rubisco S 567908–568264 bp) were located upstream of the genes encoding RuBisCO activation proteins (LT988_02760, *CbbQ* 568387–569193 bp and LT988_02770, *CbbO* 569671–572049 bp), with the 23Sr gene (LT988_02765, 569275–569637 bp) encoding the four-helix bundle protein located in-between. The transcriptional regulator of the RuBisCO operon (LT988_RS02745, *CbbR*) is located within 566015–565080 bp. This gene has an inverted orientation relative to the other RuBisCO genes.

In another case, based on the classical RAST 2 annotation, the RuBisCO structural genes (LT988_RS07075, *CbbL*, 1573243–1574658 bp and LT988_RS07080, *CbbS*, 1574821–1575165 bp) were located upstream of the genes encoding carboxysome proteins (carboxysome shell proteins *CsoS2* (LT988_07085) and *CsoS3* (LT988_07090), putative carboxysome peptides A and B (LT988_07095 and LT988_07100, respectively), two carboxysome shell proteins *CsoS1* (LT988_07105 and LT988_07110)), and bacteriopheophytin (suspected to be related to carboxysome, LT988_07115). The transcription regulator of the RuBisCO operon is located at some distance (LT988_07130, 1582292–1583305 base pairs). Carboxysomes are organelle-like protein microcompartments found in cyanobacteria and many chemoautotrophic bacteria. They elevate the efficiency of carbon fixation by abolishing oxygen access to RuBisCO, located under the protein envelope, as well as via the mechanism of carboanhydrase-dependent CO_2_ concentration [47,48]. In *Alc. vinosum* DSM 180^T^, low CO_2_ caused a preferential expression of the RuBisCO form flanked by carboxysomal genes, compared to the second RuBisCO form that dominates at high CO_2_. This is considered to support the role of the carboxysome-dependent CO_2_ concentration mechanism in this bacterium [32]. However, there is still no data regarding the existence of carboxysomes in *T. boborovii* BBS. Nevertheless, analysis of the genomes of *Alc. vinosum* DSM 180^T^ and *T. bogorovii* BBS using RAST ver. 2.0 revealed 15 and 23 genes associated with the carboxysome system, 19 and 22 genes associated with photorespiration (involving RuBisCO oxygenase activity), and 13 and 17 genes associated with the Calvin–Benson–Bassham cycle, respectively.

Thus, the analysis of the genomic sequence of *T. bogorovii* BBS allowed us to detect several genes for different forms of RuBisCO and complemented the data obtained by Turova et al., who found only part of the single gene encoding the green-like RuBisCO form I in this bacterium using oligonucleotide primers specific to different types and forms of RuBisCO genes [31]. Further studies are needed to confirm the expression activity of the identified genes and to determine their physiological roles in metabolism in *T. bogorovii* BBS.

#### 3.3.3. Heterotrophy

*T. bogorovii* BBS assimilates organic substrates during photoheterotrophic growth and can utilize them both as electron donors and also as carbon sources. The main metabolic pathways of carbon metabolism are the tricarboxylic acid cycle, the Entner–Doudoroff pathway, and the Embden–Meyerhof–Parnas pathway.

Organic acids and substrates metabolized through acetyl-CoA are assimilated via the tricarboxylic acid cycle (TCA cycle). *T. bogorovii* BBS is categorized as a purple bacterium, in which neither tricarboxylic acid cycle functions, by its ability to consume few organic compounds from the growth medium and usually only as additional carbon sources. However, according to the results of KEGG-Pathway analysis of the complete genome of *T. bogorovii* BBS and the genome of *Alc. vinosum* DSM 180T, both bacteria possess genes of all enzymes necessary for the functioning of the tricarboxylic acid cycle [7].

The glyoxylate cycle genes required for the assimilation of acetate as the only source of organic compounds (isocitrate lyase (EC: 4.1.3.1) and malatesynthase (EC: 2.3.3.9)) are present in the genome of *T. bogorovii* BBS (LT988_08090 and LT988_06350) as well as in the genome of *Alc. vinosum* DSM 180^T^ (isocitrate lyase, Alvin_1848 and malate synthase G, Alvin_2606).

The lack of beneficial effect after adding certain substrates to an inorganic medium can be explained by the lack of their intracellular transport. The revealed contradictions are the basis for further studies of the functional activity of genes of the considered metabolic pathways at the levels of transcription and proteomics.

Experimental research has shown that cell yield is elevated under the light if the inorganic growth medium is supplemented with acetate, pyruvate, lactate, glycerol, or glucose (0.1%) [31]. Meanwhile, adding the compounds that serve as TCA cycle intermediates (2-ketoglutarate, fumarate, succinate, or malate) and their precursors (propionate) induces a slight gain in the biomass, representing a difference between *T. bogorovii* BBS and *Alc. vinosum* DSM 180^T^ [32]. Meanwhile, the biomass yield is not affected by such organic compounds as formate, butyrate, fructose, galactose, lactose, sorbose, arabinose, rhamnose, xylose, mannose, maltose, sucrose, methanol, ethanol, butanol, propanol, isopropanol, mannitol, sorbitol, and dulcitol. Some compounds (benzoic acid, isobutanol, and amyl and isoamyl alcohols) are known to inhibit the growth of *T. bogorovii* BBS [31]. 

Earlier works have shown that *T. bogorovii* BBS has an open reductive TCA cycle (rTCA) typical of bacteria lacking the full set of TCA enzymes [7,49]. Essentially, rTCA functions as a reverse oxidative TCA employed by bacteria as a pathway for autotrophic CO_2_ fixation and acetate assimilation. The full turn of the cycle under autotrophic conditions leads to the fixation of four CO_2_ molecules with the formation of one oxaloacetate molecule. Two irreversible TCA cycle reactions in the reductive cycle are catalyzed by the alternative enzymes. The formation of 2-oxoglutarate from succinyl-CoA and CO_2_ is catalyzed by 2-oxoglutarate synthase (EC: 1.2.7.3 1.2.7.11). The irreversible citrate synthase can be substituted by the reversible ATP-dependent citrate lyase (EC: 2.3.3.8) [50] or by two sequentially functioning enzymes: citryl-CoA synthetase (EC: 6.2.1.18) and citryl-CoA ligase (EC: 4.1.3.34) [51]. Analysis of the *T. bogorovii* BBS genome with KEGG revealed one of these key genes encoding 2-oxoglutarate synthase (EC: 1.2.7.3 1.2.7.11; LT988_18060, LT988_18065). However, the enzymes required to replace the irreversible citrate synthase involved in the oxidative TCA cycle have not been identified yet; therefore, this pathway has been termed the open rTCA cycle. The genes encoding the key rTCA cycle enzymes are absent in *Alc. vinosum* DSM 180^T^ as well. The genes encoding the enzymes of TCA and rTCA cycles in *T. bogorovii* BBS are listed in Table 5.

Conversion of carbohydrates to phosphoenolpyruvate, pyruvate, and acetyl-CoA occurs via the Entner–Doudoroff and Embden–Meyerhof–Parnas pathways. According to the KEGG Annotation, the genomes of *T. bogorovii* BBS and *Alc. vinosum* DSM 180^T^ contain the genes of glycolytic enzymes (Embden–Meyerhof pathway) and enzymes involved in pyruvate oxidation and gluconeogenesis (Table 6). 

The Entner–Doudoroff pathway has no genetic potential for function in the bacteria when considered according to the KEGG Pathway database. This confirms the results previously obtained when studying *T. bogorovii* BBS at the physiological level.

According to KEGG, the genes required for the pentosophosphate pathway function are present in the genome of *T. bogorovii* BBS (Table 7).

It should be noted that *T. bogorovii* BBS has the gene LT988_13700 encoding D-glucose permease of the phosphotransferase system (PTS, which is absent in the genome of *Alc. vinosum* DSM 180^T^). This enzyme (EC 2.7.1.199) is a component (known as enzyme II) of the phosphoenolpyruvate (PEP)-dependent sugar transporting PTS.

Further research is required to confirm the functions of the proteins encoded by the identified genes.

#### 3.3.4. Hydrogenases

Hydrogenases belong to a class of oxidoreductases that can catalyze molecular hydrogen activation. They are widely distributed in archaea, bacteria, and some unicellular eukaryotes. These enzymes are involved in highly diverse biological processes [52] where they perform different functions. In some cases, the organisms produce hydrogen to remove an excess of reducing equivalents; in other cases, they uptake hydrogen and utilize it as an electron source. Hydrogen uptake hydrogenases are used by nitrogen-fixing microorganisms to utilize the hydrogen generated by the nitrogenase system. A separate group of sensor hydrogenases is involved in the transcription induction of hydrogen uptake hydrogenases [52,53].

Following the current classification, hydrogenases are divided into 38 classes: 29 subclasses of Ni-Fe hydrogenases, 8 subtypes of Fe-Fe hydrogenases, and monophyletic Fe hydrogenases [54]. *T. bogorovii* BBS is known to have five genes encoding Ni-Fe hydrogenases belonging to different groups and performing different functions (HupSL, HydSL, hupUV, and two Hox hydrogenases) [11,12,13,55,56,57]. Membrane-bound-hydrogen-uptake hydrogenases in *T. bogorovii* BBS comprise HupSLC and HydSL hydrogenases.

HupSL hydrogenase is involved in the uptake of the exogenous and endogenous molecular hydrogen with the transfer of electrons to the ubiquinone pool. This enzyme is also known to participate in the reverse uptake of hydrogen produced by nitrogen fixation [52]. This hydrogenase is thought to ensure bacterial growth under photoautotrophic conditions as well as chemolithotrophic growth under microaerophilic conditions in the dark [10,57]. *HupS* (LT988_02910) and *hupL* (LT988_02915) encode the small and large subunits of HupSLC hydrogenase, respectively. *HupC* (LT988_02920) is the cytochrome of the *b* type. The genome of *T. bogorovii* BBS also contains the genes *hupDHI* required for the biosynthesis of HupSLC hydrogenase [55]: LT988_02925 (Hydrogenase expression/formation protein HupD), LT988_02930 *hupH* (comparison of the nucleotide sequence of *hupH* from the AY837591.1 (GenBank) with the sequence from the whole genome of *T. bogorovii* BBS using tBlastn revealed the differences reflected in 92% identity of amino acid sequences), and LT988_02935 (Rubredoxin Gene *hupI*).

Hyd hydrogenase can reversibly catalyze the reaction towards hydrogen production as well as hydrogen uptake [58,59]. However, to date, the only known major physiological cellular function of Hyd is to catalyze the reduction of elemental sulfur with hydrogen uptake in the dark [10]. HydSL is encoded by LT988_03130 (HydS subunit) and LT988_03145 (HydL subunit). The structural genes encoding the large and small subunits are separated by two open reading frames (LT988_03135 and LT988_03140) called *isp1* and *isp2*. The Isp1 protein encoded by the gene with the same name is a heme-containing transmembrane protein of *b* type, whereas the sequence of Isp2 demonstrates a substantial level of similarity with heterosulfide reductases [11].

Despite the presence of HupUV genes (LT988_05045 and LT988_05050) in the genome of *T. bogorovii* BBS, this bacterium does not express this enzyme that commonly serves as a hydrogen sensor in other bacteria and is involved in the synthesis of HupSL hydrogenases [60].

Soluble hydrogenases belonging to the HoxEFUYH type of Ni-Fe hydrogenases and involved in NAD+ reduction or NADH oxidation, have been shown experimentally [13,61,62]. The activity of Hox hydrogenases is closely linked to sulfur metabolism. Elemental sulfur serves as an electron donor for hydrogen production under light [10]. The Hox1 hydrogenase consists of the HoxYH hydrogenase (LT988_09435 and LT988_09445) and HoxFU diaphorase (LT988_09425 and LT988_09430). HoxE (LT988_09420) is a fifth subunit that is thought to be involved in an electron transfer, followed by a gene (LT988_09450) encoding the hydrogenase maturation protein, namely HoxW.

In *T. bogorovii* BBS, Hox2 hydrogenase, which was later identified and characterized [13], is encoded by the genes Hox2YH (LT988_16220 and LT988_16225), Hox2FU (LT988_16210 and LT988_16215), and Hox2W (LT988_18265).

HydSL, HupSLC, and Hox1 enzymes have been identified and described in *Alc. vinosum* DSM 180^T^ [32]; however, in contrast to *T. bogorovii* BBS*,* this bacterium lacks the genes of the sensor hydrogenase HupUV. The *Alc. vinosum* DSM 180^T^ genome contains the genes of the other two hydrogenases. The sequences of one of the hydrogenases (Alvin_0807 to Alvin_0810) show a high degree of similarity with the hydrogenase/sulforeductases (EC: 1.12.98.4) of *Thermodesulfovibrio yellowstonii* DSM 11347 and *Chlorobium tepidum* [32]. Searching for the genes encoding this enzyme in the genome of *T. bogorovii* BBS using tBlastn revealed that the highest degree of similarity was between the sequences of the sulfhydrogenase and Hox2 hydrogenase genes.

According to the KEGG Orthology annotation, *Alc. vinosum* DSM 180^T^ has another hydrogenase, Ech hydrogenase, which catalyzes H_2_-dependent reduction of 2[4Fe-4S] ferredoxin in *Methanosarcina barkeri* as well as hydrogen generation with the reduced ferredoxin as an electron donor [63]. This hydrogenase consists of the A (Alvin_0338, function number according to the KEGG Orthology: K14086), B (Alvin_0337; K14087), C (Alvin_0331; K14088),D (Alvin_0330; K14089), E (Alvin_0329; K14090), and F subunits (Alvin_0328; K14091). 

Thus, analysis of the bacterial genome searching for known forms of hydrogenases revealed the presence of hydrogenase genes previously demonstrated experimentally.

#### 3.3.5. Chemotrophic Metabolism

Upon photoautotrophic growth, sulfide, thiosulfate, sulfur, or H_2_ are utilized as electron donors. Chemoautotrophic growth in the dark under anaerobic conditions is possible due to thiosulfate used as a sulfur source as well as an electron donor and energy source for CO_2_ assimilation [2].

*T. roseopersicina* and *T. bogorovii* BBS are known to grow under chemolithotrophic conditions utilizing thiosulfate as an electron donor [2]. It should be noted that, in theory, CO_2_ fixation involving RuBisCO can occur in the presence of oxygen, since its synthesis is continued in the presence of O_2_ in both *Thiocapsa* species. It might be related to the presence of the RuBisCO carboxysome form described in the ‘Autotrophy and RuBisCO’ subparagraph.

Similar to *Alc. vinosum* DSM 180^T^ [32], the genome of *T. bogorovii* BBS contains the genes encoding both oxidases required for the chemotrophic growth of the bacteria in the presence of oxygen. Cytochrome *bd* oxidase is encoded by the genes LT988_17870 (annotated as the ubiquinol oxidase subunit I, its amino acid sequence exhibits 85% identity with the sequence of the oxidase Alvin_2499 in *Alc. vinosum* DSM 180^T^), LT988_17865 (annotated as the cytochrome *d* ubiquinol oxidase subunit II, 80% identity with Alvin_2500 in *Alc. vinosum* DSM 180^T^), and LT988_17860 (annotated as the cytochrome *bd*-I oxidase subunit CydX, 73% identity with Alvin_2501 from *Alc. vinosum* DSM 180^T^). Cytochrome *bd* oxidase from *Alc. vinosum* DSM 180^T^ is known to function mostly under microanerobic conditions, removing toxic oxygen generated during nitrogen fixation [64].

Cytochrome cbb3 oxidase from *T. bogorovii* BBS is encoded by the genes located between LT988_18880 and LT988_18925. Cytochrome cbb3 oxidase maintains its catalytic activity in the presence of low oxygen and is capable of proton translocating [32]. We demonstrated the presence of genes responsible for chemotrophic metabolism. Further research is required to confirm the functions of the proteins encoded by these genes.

#### 3.3.6. Nitrogen Metabolism

According to genome annotation (Subsystem Technology (RAST; version 2.0) online genome analysis software), the fraction of genome involved in nitrogen metabolism in *T. bogorovii* BBS is 1.8 times greater than in *Alc. vinosum* DSM 180^T^ (Table 2). Both bacteria are known to be diazotrophs [32,65]. Their ability to fix molecular nitrogen is provided by a Mo-containing-bicomponent complex of the metalloenzyme nitrogenase encoded by *nifHDK* genes. The genomic DNA of *T. bogorovii* BBS contains all the genes of the nitrogenase complex: LT988_07970 (NifH, nitrogenase iron protein), LT988_07975 (NifD, nitrogenase molybdenum-iron protein alpha chain), and LT988_07980 (NifK, nitrogenase molybdenum-iron protein beta chain). Similar to *Alc. vinosum* DSM 180^T^ [32,65], in *T. bogorovii* BBS genes associated with nitrogen fixation are located in various genomic regions and organized in genetic clusters.

*T. bogorovii* BBS can utilize ammonium salts, urea, peptone, casein hydrolyzate, and arginine as nitrogen sources. However, it cannot grow in the presence of alanine or hydroxylamine [31]. Furthermore, *T. bogorovii* BBS does not grow when supplemented with glutamic acid or KNO_3_. According to the online analysis tools and KEGG Pathway database, this bacterium has a shortage of genes involved in assimilatory nitrate reduction (ferredoxin-nitrate reductase [EC:1.7.7.2]; nitrate reductase (NAD(P)H) [EC:1.7.1.1 1.7.1.2 1.7.1.3]; assimilatory nitrate reductase catalytic subunit [EC:1.7.99.-], catalyzing formation of nitrite from nitrate; and ferredoxin-nitrite reductase [EC:1.7.7.1], nitrite reductase (NAD(P)H) [EC:1.7.1.4]. However, it has a gene encoding the nitrite reductase [NAD(P)H] small subunit [EC:1.7.1.4] *nirD*, LT988_07740). The bacterium under scrutiny contains some genes required for denitrification (nitrite reductase (NADH) large subunit [EC:1.7.1.15], nitrite reductase (cytochrome *c*-552) [EC:1.7.2.2]), and nitrification (methane/ammonia monooxygenase subunit A [EC:1.14.18.3 1.14.99.39] and nitrate reductase/nitrite oxidoreductase, alpha subunit [EC:1.7.5.1 1.7.99.-]). At the genomic level, this bacterium has the potential for the first reaction of the dissimilatory nitrate reduction, conversion of nitrate into nitrite by nitrate reductase/nitrite oxidoreductase, alpha subunit [EC:1.7.5.1 1.7.99.-] (LT988_06635 encoding respiratory nitrate reductase subunit gamma; and LT988_07690 encoding nitrate reductase cytochrome *c*-type subunit). Analysis of the *Alc. vinosum* DSM 180^T^ genome revealed the lack of the genes involved in the above processes mentioned above [32].

Thus, we have clearly demonstrated the presence of genes responsible for nitrogen metabolism, experimentally discovered here based on previous physiological data. Further studies are needed to confirm the functions of the proteins encoded by the identified genes.

#### 3.3.7. Sulfur Metabolism

*T. bogorovii* BBS grows only in the presence of S_0_, S^2–^, or S_2_O_3_- [31]. It does not utilize cysteine and methionine as sulfur sources. Sulfide and thiosulfate are oxidized to sulfate via the formation of elemental sulfur accumulated in cells as granules. There are several known pathways for utilizing sulfur compounds as electron donors and acceptors for energy acquisition.

In contrast to *Alc. vinosum* DSM 180^T^ [32], *T. bogorovii* BBS is not capable of assimilatory sulfate reduction [31]. This is confirmed by the results of KEGG Pathway genome analysis. This bacterium lacks the genes encoding the enzymes of phosphoadenosine phosphosulfate reductase [EC:1.8.4.8 1.8.4.10], sulfite reductase (NADPH) [EC:1.8.1.2], and alternative ferredoxin-sulfite reductase [EC:1.8.7.1].

##### Thiosulfate Oxidation

Thiosulfate oxidation can occur in the periplasm with the formation of tetrathionate (S_4_O_6_^2−^) or in the periplasmic multi-enzyme sulfur oxidation system (Sox system) [66]. The main enzyme of the tetrathionate pathway in *Alc. vinosum* DSM 180^T^ functions as tetrathionate reductase (TsdA, Alvin_0091 [67]). The electron transfer from TsdA to the photosynthetic or respiratory ETC upon thiosulfate oxidation involves the TsdB protein (diheme cytochrome *c*4 encoded by Alvin_2879 in *Alc. vinosum*) and possibly protein Alvin_2880 [66]. Another possible acceptor of electrons from TsdA in the purple sulfur bacteria is the high potential protein HiPIP (350 mV). In the *T. bogorovii* BBS genome, there is no gene encoding TsdA or the alternative archaeal enzyme AoxDA (thiosulfate/quinone oxidoreductase, which is absent in phototrophic prokaryotes).

The second pathway of thiosulfate oxidation is mediated by the Sox system involved in the disproportionation of thiosulfate with the formation of sulfate and molecular sulfur [66]. In this pathway, protein-bound sulfur atoms undergo oxidation. The genes involved in the Sox pathway are present both in *Alc. vinosum* DSM 180^T^ [32] and *T. bogorovii* BBS *(*Table 8). LT988_16035 (SoxY), LT988_16040 (SoxZ), LT988_24235 (SoxB), LT988_24240 (SoxX), LT988_24245 (SoxA), and also LT988_24250 (61% identity with the amino acid sequence of the SoxXA-binding protein from *Alc. vinosum* DSM 180^T^ (SoxK, Alvin_2170)); LT988_24255 and LT988_09460 (61% and 43% identity with the periplasmic sulfur transferase from *Alc. vinosum* DSM 180^T^ (SoxL, Alvin_2171)). SoxL serves as an alternative to the SoxCD proteins and is typical of bacteria that do not accumulate sulfur. The SoxCD encoding genes are absent both in *T. bogorovii* BBS and *Alc. vinosum* DSM 180^T^.

##### Sulfide Oxidation

Several enzymes can oxidize sulfides: sulfide/quinone oxidoreductase (SQR), flavocytochrome *c* sulfide dehydrogenase (FccAB), and the Sox system in bacteria that do not accumulate sulfur [66].

The described SQRs are single-subunit flavoproteins bound to the cytoplasmic membrane. They are classified into six types based on structural data. SQRA and SQRE types are not found in the purple sulfur bacteria, SqrF and SqrD proteins are widely distributed among the *Chromatiaceae* purple sulfur bacteria, and the ScrB type is common among the *Ectothiorhodospirilaceae* family members [66]. The primary product of the SQR reaction is polysulfide.

*T. bogorovii* BBS has the genes LT988_02950 and LT988_15980, annotated as the FAD-dependent oxidoreductase (Table 8, reaction 6) according to GenBank and homologous to the genes from *Alc. vinosum* DSM 180^T^ encoding SqrD and SqrF proteins, respectively. In contrast to *Alc. vinosum* DSM 180^T^, *T. bogorovii* BBS lacks the genes Alvin_1196 and Alvin_1197 that are located downstream of SqrF and are possibly responsible for attaching the enzyme to the membrane in *Alc. vinosum* DSM 180^T^ [32].

Sulfide oxidation can involve the flavocytochrome *c* sulfide reductase in the periplasmic space; however, the physiological role of this enzyme has not been clarified yet (the knockout bacteria oxidize sulfide with the same rates [32], whereas some species of green and purple sulfur bacteria do not produce the proteins). *T. bogorovii* BBS has two genes, LT988_00625 and LT988_00620 (Table 8, Nos. 7,8), that are homologous to the genes encoding the FccB and FccA subunits of flavocytochrome *c* sulfidereductase FccAB from *Alc. vinosum* DSM 180^T^.

##### Oxidizing Elemental Sulfur

Elemental sulfur is almost insoluble in water, and it has not been fully understood how phototrophic organisms bind, activate, and uptake this substrate [66]. Exogenous elemental sulfur mainly consists of S_8_ rings, chains of polymeric sulfur, and traces of S_7_ rings. The uptake of elemental sulfur by *Alc. vinosum* DSM 180^T^ seems to require the direct interaction of cells with sulfur and, although unlikely, the action of secreted compounds towards the substrate outside the cell.

The purple sulfur bacteria of the *Chromatiaceae* family accumulate molecular sulfur in the periplasmic space in the form of globules surrounded by the protein coat. In *Alc. vinosum* DSM 180^T^, the protein coat is a monolayer consisting of four sulfur globule proteins, SgpABCD, that perform an exclusively structural function and that are required for sulfur formation and deposition. SgpA (Alvin_1905, annotated as Sulfur globule protein CV1) and SgpB (Alvin_0358, annotated as Sulfur globule protein CV2) are similar, have a weight of 10.5 kDa, and can partially substitute for each other. The SgpC protein encoded by Alvin_1325 (annotated as Sulfur globule protein CV3) is important for globule expansion. SgpD (Alvin_2515) is the most common protein of these globules, as shown by the proteomic studies. According to the sequencing genomic data, Sgp proteins are present in all members of purple sulfur bacteria from the *Chromatiaceae* family in variable combinations [32] and absent in the members of the *Ectothiorhodospirilaceae* family. Earlier, the protein sequences of two large Sgps isolated from *Alc. vinosum* strain ATCC 17899 (10.5 kDa) were demonstrated to be similar to one of the two isolated Sgp proteins from the *T. roseopersicina* strain SMG219. Likewise, the sequences of small Sgp proteins from these bacteria (8.5 and 8.7 kDa, respectively) were found to show a certain amount of similarity as well [68]. Three genes annotated as sulfur globule proteins were discovered in *T. bogorovii* BBS. They are LT988_04675, encoding sulfur globule protein CV3; LT988_09360, encoding sulfur globule protein CV1; LT988_19155, encoding sulfur globule protein CV1 LT988_19155; and two more genes that are classified as sulfur globule family proteins (LT988_08020 and LT988_19100). No associated KEGG Orthology function has been identified for these genes.

In *Alc. vinosum*, sulfur is present in the globules in the form of mono- and bis-organylsulfanes [69]. The sulfur accumulated in the globules can undergo oxidation after being activated and then translocated to the cytoplasm with a vehicle perthiol molecule. There are two known pathways of sulfur oxidation: the Dsr system (which involves reversible dissimilatory sulfite reductase DsrAB) and the sulfur oxidation pathway involving the system of enzymes similar to heterosulfide reductase [66].

Low-molecular-weight organic persulfides such as glutathion persulfide can serve as vehicle molecules translocating sulfur from periplamic or extracellular deposits to the cytoplasm [70]. As yet, the pathway mediating formation of possible molecules in persulfide vehicles has not been studied; it is not known whether there are specific enzymes and transporters involved in this process. In *Alc. vinosum* DSM 180^T^, sulfur from the globules is translocated to the active center of sulfite reductase DsrAB via the cascade of persulfide intermediates located on the rhodanese, TusA, and probably DsrE2A, DsrE, and DsrC [61]. Along with SoxL, six other genes that can encode rhodanese in *Alc. vinosum* DSM 180^T^ have been annotated: Alvin_0258, Alvin_0866, Alvin_0868, Alvin_1587, Alvin_2599, and Alvin_3028. Currently, the role of these proteins in the dissimilatory sulfur metabolism is not clear. The *T. bogorovii* BBS genome contains seven genes encoding the products annotated as rhodanese-like domain-containing protein (LT988_07780, LT988_09460, LT988_12845, LT988_16050, LT988_19985, LT988_23355, and LT988_24255), some of which show similarity with rhodanese genes from *Alc. vinosum* DSM 180^T^. None of these proteins from *T. bogorovii* BBS have been assigned a function according to KEGG Orthology. Previously, the product of the Rhd_2599 gene (Sulfurtransferase Alvin_2599, rhodanese-like protein) has been experimentally shown to serve as a sulfur vehicle and to participate in sulfur transfer involved in oxidative-dissimilatory sulfur metabolism [71]. In *Alc. vinosum,* this protein provides sulfur mobilization and the transfer of sulfur from a low-molecular-weight thiol (probably from glutathion) to the TusA protein (Alvin_2600); TusA serves as a sulfur donor for DsrEFH (genes Alvin_1253, Alvin_1254, and Alvin_1255), which in its turn perform the persulfation of DsrC (Alvin_1256); persulfated DsrC is likely to serve as a direct substrate for reverse sulfite reductase DsrAB. Rhd_2599, TusA, and DsrE2 in *Alc. vinosum* DSM 180^T^ have been shown to bind sulfur atoms via conserved cysteine residues.

The Dsr proteins of *Alc. vinosum* DSM 180^T^ are encoded by the same gene cluster, *dsrABEFHCMKLJOPNRS*, which is located downstream of Alvin_1251 and upstream of Alvin_1265 and Alvin_2601 (DsrE2). *T. bogorovii* BBS has genes *dsrABEFHCMKLJOPNRS* (located in from LT988_06665 to LT988_06595 in the genome), but the only genes annotated in the same manner are *dsrA* (LT988_06665) and *dsrB* (LT988_06660); the other genes were identified using the tBlastn algorithm (Table 9). Furthermore, the genome contains several more genes annotated as TusE/DsrC/DsvC family sulfur relay protein (LT988_03825, LT988_03875, LT988_06640, LT988_06690, LT988_16185, LT988_16425, and LT988_19515) according to GenBank.

**Figure 3:**
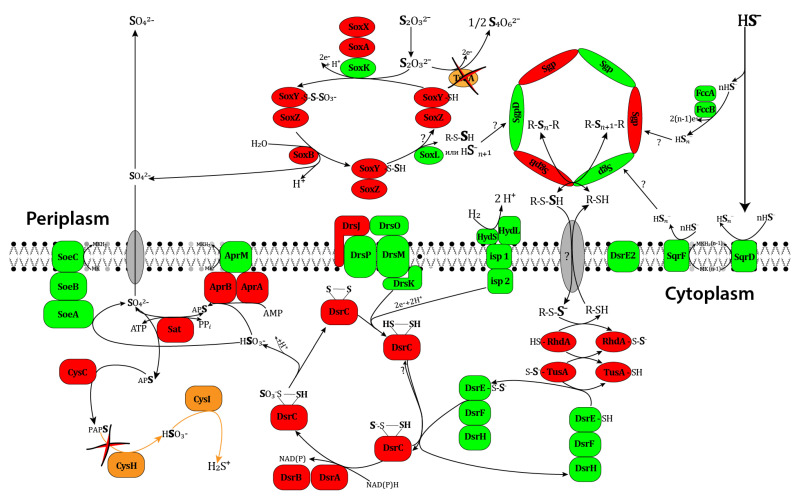
The scheme of sulfur metabolic pathways in purple non-sulfur bacteria. The proteins encoded by genes from the annotated sequence of *T. bogorovii* BBS with names different from those in *Alc. vinosum* DSM 180^T^ are shown in red; the proteins with the same names are shown in green; the proteins absent in *T. bogorovii* BBS are shown in brown; currently unidentified proteins are shown in gray.

The results obtained were compared with the data on the possible pathways of sulfate oxidation in purple sulfur bacteria summarized in the reviews [61]. Figure 3 shows the scheme demonstrating the reactions of sulfur metabolism that were revealed in *T. bogorovii* BBS at a genomic level.

## 4. Conclusions

The phototrophic purple sulfur bacterium *T. bogorovii* BBS belonging to the *Chromatiaceae* family was isolated from the White Sea Estuary in 1969 [1]. In this work, we have obtained, assembled, and published the whole genome of *T. bogorovii* BBS (GenBank: CP089309.1). Analysis of the genome sequence allowed us to validate the systematic position of the bacteria and to confirm the appropriateness of classifying it as a new typical species of the genus *Thiocapsa*, as proposed by Tourova et al. [31].

Some genes involved in pigment synthesis in T. roseopersicina BBS have been described previously, but identification of genes outside the genomic DNA fragment examined by the authors was required. Through mining the whole genome of *T bogorovii* sequence, we found eleven additional genes coding proteins involved in pigment biosynthesis.

We employed the functional gene annotation capabilities of the KEGG database, the Subsystem Technology software for genome analysis (RAST; version 2.0), performed a comparative analysis with the genome of the purple bacterium *Alc. vinosum* DSM 180^T^, and manually searched for several genes using the tBlastn algorithm. As a result, we both located and functionally characterized the genes in the genomic DNA that determine the function of central metabolic pathways in *T. bogorovii* BBS. Two copies of the RuBisCO green-like form I genes allow RuBisCO-mediated CO_2_ fixation in this bacterium. The genes are located far apart and have different neighborhoods. In one case, the gene is flanked by the genes encoding RuBisCO activation proteins. In the other case, the genes encoding carboxysome proteins and the regulator of the RuBisCO operon transcription are located downstream of the RuBisCO structural genes. Along with concentrating CO_2_, carboxysomes protect RuBisCO from O_2_, as O_2_ can compete with CO_2_. However, there is no information in the pertinent literature about carboxysomes in bacterial cells. The genome of the bacterium under scrutiny also contains a gene belonging to the type IV RuBisCO-like proteins that is not involved in CO_2_ fixation. According to the KEGG Pathway database, *T. bogorovii* BBS has all of the genes encoding the enzymes that are required for the TCA and glyoxylate cycles.

In earlier works, *T. bogorovii* BBS was demonstrated to have the reductive TCA cycle, which is supported by the genomic data in this present work. The cycle is open due to the absence of the enzymes required to substitute for irreversible citrate synthase from the oxidizing TCA cycle. 

The *T. bogorovii* BBS genome contains the genes from the Embde–Meyerhof–Parnas pathway, pyruvate oxidation, and gluconeogenesis for the synthesis and conversion of carbohydrates to phoshoenolpyruvate, pyruvate, and acetyl-CoA. The Entner–Doudoroff pathway has no genetic potential in *T. bogorovii* BBS. 

We manually searched for hydrogenase genes and determined their locations in the bacterial genome. These are in full accordance with previously published data.

We have identified the genes for the cytochromes *bd* and cbb3 of oxidases required for chemotrophic growth of the bacteria in the presence of oxygen. 

The ability to fix atmospheric molecular nitrogen is ensured by the existence of genes for the Mo-containing nitrogenase complex nifHDK. 

*T. bogorovii* BBS lacks some of the genes required for the assimilatory nitrate reduction, denitrification, and nitrification. However, it has the potential for dissimilatory nitrate reduction. This finding is new and not supported by previous biochemical data.

It is known that HydSL hydrogenase from *T. bogorovii* BBS can be simply immobilized on carbon electrodes with proper orientation between a distal FeS cluster and an electrode and the production of high current densities [72]. Taking into account the fact that that this hydrogenase is stable and can work even at 70°C, one can conclude that it is a very promising enzyme for use in enzymatic hydrogenase electrodes. Several schemes of HydSL hydrogenase participation in metabolism of *T. bogorovii* BBS exist [14,66,73]. All of them indicate that HydSL hydrogenase in purple bacteria is closely connected with sulfur metabolism but, unfortunately, none of them took into account all valid experimental data. We have genetically analyzed the sulfur metabolism of *T. bogorovii* BBS and compared it with the most studied sulfur metabolism of *Alc. vinosum* [66]. This knowledge is important for understanding the mechanisms involved in electron transfer between hydrogenases and native partners. The automatic annotation of the whole genome of the bacteria *T. bogorovii* BBS was refined and the genomic positions of several studied genes, including those involved in sulfur metabolism, were localized. The bacterium has no genetic potential for assimilatory sulfate reduction, but it has the genes required for dissimilatory sulfate reduction and thiosulphate oxidation by the SOX-complex. Figure 3 shows a scheme demonstrating the reactions of sulfur metabolism revealed in *T. bogorovii* BBS at a genomic level. 

The functional potential of central metabolic pathways identified at the genome level in the bacterium undoubtedly requires further experiments to confirm their activity at the transcriptional and enzymatic levels. Some gene sequences are present in the *T. bogorovii* BBS genome in several copies. Further research is required to confirm the functions of the proteins encoded by the identified genes. 

## Figures and Tables

**Table 1:** Comparative genome statistics for the purple sulfur bacteria *T. roseopersicina* BBS and *Alc. vinosum* DSM 180^T^.

Feature	*T. roseopersicina* BBS	*Alc. vinosum* D^T^ (DSM 180^T^)
GenBank accession number(s)	NZ_CP089309.1	CP001896; CP001897; CP001898
Genome size (bp)	5,649,927	3,669,074
Number of replicons	1	3
Extrachromosomal elements (size)	0	2 (102 kbp; 40 kbp)
Percent G + C content %	63.94	64.2
Total number of genes	5036	3327
Protein-encoding genes (% of total)	4854 (96.4)	3217 (96.7)
Total RNA genes	58	64
tRNAs	48	51
rRNA genes/operons	2, 2, 2 (5S, 16S, 23S)	9/3
ncRNAs	4	4
Putative pseudogenes	124	46
CRISPR arrays	1	3

**Table 2:** The level of similarity between the *T. roseopersicina* BBS genome and other typical species of the *Thiocapsa* genus *.

Name of Organism	ANI Value, %	DDH, %
*T. marina* 5811T	89.03	50.9
*T. rosea* DSM235T	86.92	38.3
*T. imhoffii* DSM21303	75.58	14.6
*T. roseopersicina* DSM217T	87.22	43.2

* Note that the recommended cut-off point of 70% DDH for species delineation corresponded to 95% ANI and 69% conserved DNA.

**Table 3:** Comparison of major gene categories * encoded by the genomes of *T. bogorovii* BBS and *Alc. vinosum* DSM 180 ^T^.

Category	*T. bogorovii* BBS		*Alc. vinosum* DSM 180 ^T^	
	No. of Genes	% Genome Content	No. of Genes	% Genome Content
Protein metabolism	316	10.33	264	10.85
Vitamins, cofactors, prosthetic groups, and photosynthetic pigments	284	9.28	249	10.23
Amino acids and derivatives	307	10.03	235	9.66
RNA metabolism	191	6.24	162	6.66
Respiration	196	6.41	178	7.31
Carbohydrate metabolism	288	9.41	183	7.52
Motility and chemotaxis	0	0	161	6.62
Cell wall structure	182	5.95	161	6.62
DNA metabolism	183	5.98	76	3.12
Membrane transport	164	5.36	102	4.19
Fatty acids, lipids, and isoprenoids	103	3.37	88	3.62
Stress response	154	5.03	107	4.4
Virulence (disease and defense)	103	3.37	77	3.2
Nucleosides and nucleotides	79	2.58	61	2.51
Phosphorus metabolism	54	1.77	43	1.8
Cell division	37	1.21	39	1.6
Sulfur metabolism	64	2.1	52	2.14
Nitrogen metabolism	89	2.91	39	1.6
Potassium metabolism	37	1.21	20	0.82

* Genome statistics for both organisms are based on computations performed by the Rapid. Annotation using Subsystem Technology (RAST; version 2.0, Annotation scheme: ClassicRAST) online genome analysis software.

**Table 4:** The genes encoding the enzymes of specific carotenoid synthesis, light-harvesting photosynthetic complexes, and genes related to bacteriochlorophyll *a* in *T. bogorovii* BBS.

№	Gene Designation and Function According to Kovács et al. [8]	Function Number, Enzyme Annotation, and Number (According to KEGG Orthology Database)	Gene ID	Gene Name and Annotation Presented in GenBank
1	-*	K08926 light-harvesting complex 1 alpha chain	LT988_08850	light-harvesting protein
2	-	K08927 light-harvesting complex 1 beta chain	LT988_08855	light-harvesting protein
3	-	K08926 light-harvesting complex 1 alpha chain	LT988_08860	light-harvesting protein
4	-	K13992 photosynthetic reaction center cytochrome c subunit	LT988_08865	photosynthetic reaction center cytochrome c subunit
5	-	K08929 photosynthetic reaction center M subunit	LT988_08870	*pufM*; photosynthetic reaction center subunit M
6	-	K08928 photosynthetic reaction center L subunit	LT988_08875	*pufL*; photosynthetic reaction center subunit L
7	-	K08926 light-harvesting complex 1 alpha chain	LT988_08880	light-harvesting protein
8	-	K08927 light-harvesting complex 1 beta chain	LT988_08885	light-harvesting protein
9	-	no KO assigned	LT988_08890	pseudogene
10	-	K11335 3,8-divinyl chlorophyllide a/chlorophyllide a reductase subunit Z [EC:1.3.7.14 1.3.7.15]	LT988_08895	*bchZ*; chlorophyllide a reductase subunit Z
11	-	K11334 3,8-divinyl chlorophyllide a/chlorophyllide a reductase subunit Y [EC:1.3.7.14 1.3.7.15]	LT988_08900	*bchY*; chlorophyllide a reductase subunit Y
12	*bchX*, Bacteriochlorophyllide reductase subunit	K11333 3,8-divinyl chlorophyllide a/chlorophyllide a reductase subunit X [EC:1.3.7.14 1.3.7.15]	LT988_08905	chlorophyllide a reductase iron protein subunit X
13	*bchC(T)*, 2-α-Hydroxyethyl bacteriochlorophyllide oxidase, 317 aa	K11337 bacteriochlorophyllide a dehydrogenase [EC:1.1.1.396]	LT988_08910 317 aa	*bchC*; chlorophyll synthesis pathway protein BchC
14	*crtF(G)*, Hydroxyneurosporene methyltransferase, 371 aa	K09846 demethylspheroidene O-methyltransferase [EC:2.1.1.210]	LT988_08915 371 aa	acetylserotonin O-methyltransferase
15	*crtE*, Geranylgeranyl pyrophosphate synthase, 288 aa	K13789 geranylgeranyl diphosphate synthase, type II [EC:2.5.1.1 2.5.1.10 2.5.1.29]	LT988_08920 288 aa	polyprenyl synthetase family protein
16	*crtD*, Methoxyneurosporene dehydrogenase, 498 aa	K09845 1-hydroxycarotenoid 3,4-desaturase [EC:1.3.99.27]	LT988_08925 498 aa	*crtI*; phytoene desaturase
	*crtC*, Hydroxyneurosporene dehydrogenase, 405 aa 1962948–1961731			
17	-	K09844 carotenoid 1,2-hydratase [EC:4.2.1.131]	LT988_08930 277 aa	carotenoid 1,2-hydratase
18	*orf495 (G)*, o-Succinyl-benzoic acid CoA ligase, 495 aa	K01911 o-succinylbenzoate---CoA ligase [EC:6.2.1.26]	LT988_08935 468 aa	AMP-binding protein
19	*bchJ (G)*, 4-Vinyl reductase, 208 aa	K04036 divinyl protochlorophyllide a 8-vinyl-reductase [EC:1.-.-.-]	LT988_08940 201 aa	*bchJ*; bacteriochlorophyll 4-vinyl reductase
20	*hemN*, O2-independent coproporphyrinogen III oxidase, 453 aa	K02495 oxygen-independent coproporphyrinogen III oxidase [EC:1.3.98.3]	LT988_08945 453 aa	*hemN*; oxygen-independent coproporphyrinogen III oxidase
21	*bchE*, Mg-protoporphyrin IX monomethylester oxidative cyclase subunit, 551 aa	K04034 anaerobic magnesium-protoporphyrin IX monomethyl ester cyclase [EC:1.21.98.3]	LT988_08950 551 aa	*bchE*; magnesium-protoporphyrin IX monomethyl ester anaerobic oxidative cyclase
22	*orf312*, Hypothetical membrane protein, 312 aa	no KO assigned	LT988_08955 312 aa	DUF3623 domain-containing protein
23	*orf139*, Hypothetical protein, 139 aa	no KO assigned	LT988_08960 151 aa	phosphonoacetaldehyde hydrolase
24	*orf218(G)*, Hypothetical membrane protein, 218 aa	no KO assigned	LT988_08965 218 aa	PH domain-containing protein
25	*puhA*, 255 aa, Photosynthetic reaction center H subunit	K13991 photosynthetic reaction center H subunit	LT988_08970 255 aa	*puhA*; photosynthetic reaction center subunit H
26	*bchM*, Mg-protoporphyrin methyltranserase, 233 aa	K03428 magnesium-protoporphyrin O-methyltransferase [EC:2.1.1.11]	LT988_08975 233 aa	*bchM*; magnesium-protoporphyrin IX methyltransferase
27	*bchL*, 294 aa, Light-independent prochlorophyllide reductase iron–sulfur-ATP-binding subunit	K04037 light-independent protochlorophyllide reductase subunit L [EC:1.3.7.7]	LT988_08980 294 aa	*bchL*; ferredoxin:protochlorophyllide reductase (ATP-dependent) iron–sulfur- ATP-binding protein
28	*bchH*, AF528191.1, 1245 aa, Mg-protoporphyrin IX chelatase H subunit	K03403 magnesium chelatase subunit H [EC:6.6.1.1]	LT988_08985 1245 aa	magnesium chelatase subunit H
29	*bchB*, AF528191.1, Light-independent prochlorophyllide reductase b subunit, 258 aa,	K04039 light-independent protochlorophyllide reductase subunit B [EC:1.3.7.7]	LT988_08990 534 aa	ferredoxin:protochlorophyllide reductase (ATP-dependent) subunit B
30	-	K04038 light-independent protochlorophyllide reductase subunit N [EC:1.3.7.7]	LT988_08995	ferredoxin:protochlorophyllide reductase (ATP-dependent) subunit N
31	-	K11336 3-vinyl bacteriochlorophyllide hydratase [EC:4.2.1.165]	LT988_09000	*bchF*; 2-vinyl bacteriochlorophyllide hydratase
32	-	K04040 chlorophyll/bacteriochlorophyll a synthase [EC:2.5.1.62 2.5.1.133]	LT988_09010	*chlG*; chlorophyll synthase ChlG
33	-	K10960 geranylgeranyl diphosphate/geranylgeranyl-bacteriochlorophyllide a reductase [EC:1.3.1.83 1.3.1.111]	LT988_09015	geranylgeranyl diphosphate reductase
34	-	no KO assigned	LT988_21295	photosynthetic reaction center cytochrome c subunit

* indicates that the information is absent.

**Table 5:** The genes encoding the enzymes of the TCA cycle (reactions 1–13), unique enzymes of the rTCA cycle (reactions, 14,15,16), and the glyoxylate cycle (reactions 17,18) in *T. bogorovii* BBS.

№	Function Number, Enzyme Annotation, and Number (According to KEGG Orthology Database)	Gene ID	Gene Name and Annotation Presented in GenBank
1	K01647 citrate synthase [EC:2.3.3.1]	LT988_13005	citrate synthase
1	K01647 citrate synthase [EC:2.3.3.1]	LT988_17095	citrate synthase
2, 3	K01681 aconitate hydratase [EC:4.2.1.3]	LT988_17115	*acnA*; aconitate hydratase AcnA
4, 5	K00031 isocitrate dehydrogenase [EC:1.1.1.42]	LT988_17130	*icd*; NADP-dependent isocitrate dehydrogenase
6, 7	K00164 2-oxoglutarate dehydrogenase E1 component [EC:1.2.4.2]	LT988_19210	2-oxoglutarate dehydrogenase E1 component
8	K00658 2-oxoglutarate dehydrogenase E2 component (dihydrolipoamide succinyltransferase) [EC:2.3.1.61]	LT988_19205	*odhB*; 2-oxoglutarate dehydrogenase complex dihydrolipoyllysine-residue succinyltransferase
9	K00382 dihydrolipoyl dehydrogenase [EC:1.8.1.4]	LT988_13640	dihydrolipoyl dehydrogenase
9	K00382 dihydrolipoyl dehydrogenase [EC:1.8.1.4]	LT988_22720	*lpdA*; dihydrolipoyl dehydrogenase
9	K00382 dihydrolipoyl dehydrogenase [EC:1.8.1.4]	LT988_05285	dihydrolipoyl dehydrogenase
6a	K00174 2-oxoglutarate/2-oxoacid ferredoxin oxidoreductase subunit alpha [EC:1.2.7.3 1.2.7.11]	LT988_18060	2-oxoacid:acceptor oxidoreductase subunit alpha
6b	K00175 2-oxoglutarate/2-oxoacid ferredoxin oxidoreductase subunit beta [EC:1.2.7.3 1.2.7.11]	LT988_18065	2-oxoacid:ferredoxin oxidoreductase subunit beta
10	K01902 succinyl-CoA synthetase alpha subunit [EC:6.2.1.5]	LT988_08455	*sucD*; succinate--CoA ligase subunit alpha
	K01903 succinyl-CoA synthetase beta subunit [EC:6.2.1.5]	LT988_08460	*sucC*; ADP-forming succinate--CoA ligase subunit beta
11	K00240 succinate dehydrogenase iron–sulfur subunit [EC:1.3.5.1]	LT988_09960	*sdhB*; succinate dehydrogenase iron–sulfur subunit
	K00239 succinate dehydrogenase flavoprotein subunit [EC:1.3.5.1]	LT988_09965	*sdhA*; succinate dehydrogenase (quinone) flavoprotein subunit [KO:K00239] [EC:1.3.5.1]
	K00241 succinate dehydrogenase cytochrome b subunit	LT988_09970	succinate dehydrogenase [KO:K00241]
11	K00240 succinate dehydrogenase iron–sulfur subunit [EC:1.3.5.1 1.3.5.4]	LT988_04620	succinate dehydrogenase iron–sulfur subunit
	K00239 succinate dehydrogenase flavoprotein subunit [EC:1.3.5.1]	LT988_04625	*sdhA*; succinate dehydrogenase flavoprotein subunit
	K00242 succinate dehydrogenase membrane anchor subunit	LT988_04630	*sdhD*; succinate dehydrogenase, hydrophobic membrane anchor protein
	K00241 succinate dehydrogenase cytochrome b subunit	LT988_04635	*sdhC*; succinate dehydrogenase, cytochrome b556 subunit
12	K01679 fumarate hydratase, class II [EC:4.2.1.2]	LT988_23020	*fumC*; class II fumarate hydratase
12	K01676 fumarate hydratase, class I [EC:4.2.1.2]	LT988_22530	fumarate hydratase
13	K00024 malate dehydrogenase [EC:1.1.1.37]	LT988_13010	*mdh*; malate dehydrogenase
13	K00116 malate dehydrogenase (quinone) [EC:1.1.5.4]	LT988_13045	FAD-dependent oxidoreductase [KO:K00116] [EC:1.1.5.4]
14	K00174 2-oxoglutarate/2-oxoacid ferredoxin oxidoreductase subunit alpha [EC:1.2.7.3 1.2.7.11]	LT988_18060	2-oxoacid:acceptor oxidoreductase subunit alpha
	K00175 2-oxoglutarate/2-oxoacid ferredoxin oxidoreductase subunit beta [EC:1.2.7.3 1.2.7.11]	LT988_18065	2-oxoacid:ferredoxin oxidoreductase subunit beta
15	K15232 citryl-CoA synthetase large subunit [EC: 6.2.1.18]	- **	-
16	K15234 citryl-CoA lyase (EC: 4.1.3.34)	-	-
17	K01637 isocitrate lyase [EC:4.1.3.1]	LT988_08090	*aceA*; isocitrate lyase
18	K01638 malate synthase [EC:2.3.3.9]	LT988_06350	malate synthase G
19	K01895 acetyl-CoA synthetase [EC:6.2.1.1]	LT988_16650	*acs*; acetate--CoA ligase

** indicates that the sequence of a gene in the genome in question is absent.

**Table 6:** The genes encoding the enzymes of the Embden–Meyerhof–Parnas pathway (1–10), gluconeogenesis (reactions 10–1 with the reaction 3 substituting the reverse reaction 11), and pyruvate oxidation (reactions 12 or 13–15) in *T. bogorovii* BBS.

№	Function Number, Enzyme Annotation, and Number (According to KEGG Orthology Database)	Gene ID	Gene Name and Annotation Presented in GenBank
1	K00845 glucokinase [EC:2.7.1.2]	LT988_04670	*glk*; glucokinase
2	K01810 glucose-6-phosphate isomerase [EC:5.3.1.9]	LT988_04445	*pgi*; glucose-6-phosphate isomerase
2	K01810 glucose-6-phosphate isomerase [EC:5.3.1.9]	LT988_25180	*pgi*; glucose-6-phosphate isomerase
3	K21071 ATP-dependent phosphofructokinase/diphosphate-dependent phosphofructokinase [EC:2.7.1.11; 2.7.1.90]	LT988_14085	6-phosphofructokinase
4	K01623 fructose-bisphosphate aldolase, class I [EC:4.1.2.13]	LT988_25065	fructose-bisphosphate aldolase class I
4	K01624 fructose-bisphosphate aldolase, class II [EC:4.1.2.13]	LT988_08070	*fba*; fructose-bisphosphate aldolase class II
5	K01803 triose-phosphate isomerase (TIM) [EC:5.3.1.1]	LT988_02660	*tpiA*; triose-phosphate isomerase
6	K00134 glyceraldehyde 3-phosphate dehydrogenase (phosphorylating) [EC:1.2.1.12]	LT988_08050	*gap*; type I glyceraldehyde-3-phosphate dehydrogenase
7	K00927 phosphoglycerate kinase [EC:2.7.2.3]	LT988_08060	phosphoglycerate kinase
8	K15634 2,3-bisphosphoglycerate-dependent phosphoglycerate mutase [EC:5.4.2.11]	LT988_13795	histidine phosphatase family protein
8	K15633 2,3-bisphosphoglycerate-independent phosphoglycerate mutase [EC:5.4.2.12]	LT988_06575	*gpmI*; 2,3-bisphosphoglycerate-independent phosphoglycerate mutase
9	K01689 enolase [EC:4.2.1.11]	LT988_19115	*eno*; phosphopyruvate hydratase
10	K00873 pyruvate kinase [EC:2.7.1.40]	LT988_01025	*pyk*; pyruvate kinase
10	K00873 pyruvate kinase [EC:2.7.1.40]	LT988_08065	*pyk*; pyruvate kinase
10	K00873 pyruvate kinase [EC:2.7.1.40]	LT988_22860	pyruvate kinase
11	K03841 fructose-1,6-bisphosphatase I [EC:3.1.3.11]	LT988_07425	class 1 fructose-bisphosphatase
12	K00172 pyruvate-ferredoxin oxidoreductase gamma subunit [EC:1.2.7.1]	LT988_13550	2-oxoacid:acceptor oxidoreductase family protein
	K00169 pyruvate-ferredoxin oxidoreductase alpha subunit [EC:1.2.7.1]	LT988_13555	hypothetical protein
	K00170 pyruvate-ferredoxin oxidoreductase beta subunit [EC:1.2.7.1]	LT988_13560	thiamine pyrophosphate-dependent enzyme
12	K03737 pyruvate-ferredoxin/flavodoxin oxidoreductase [EC:1.2.7.1 1.2.7.-]	LT988_24065	2-oxoacid:acceptor oxidoreductase family protein
	K03737 pyruvate-ferredoxin/flavodoxin oxidoreductase [EC:1.2.7.1 1.2.7.-]	LT988_24180	*nifJ*; pyruvate:ferredoxin (flavodoxin) oxidoreductase
13	K00163 pyruvate dehydrogenase E1 component [EC:1.2.4.1]	LT988_22730	*aceE*; pyruvate dehydrogenase (acetyl-transferring), homodimeric type
14	K00382 dihydrolipoamide dehydrogenase [EC:1.8.1.4]	LT988_05285	dihydrolipoyl dehydrogenase
14	K00382 dihydrolipoamide dehydrogenase [EC:1.8.1.4]	LT988_13640	dihydrolipoyl dehydrogenase
14	K00382 dihydrolipoamide dehydrogenase [EC:1.8.1.4]	LT988_22720	*lpdA*; dihydrolipoyl dehydrogenase
15	K00627 pyruvate dehydrogenase E2 component (dihydrolipoamide acetyltransferase) [EC:2.3.1.12]	LT988_22725	*aceF*; dihydrolipoyllysine-residue acetyltransferase

**Table 7:** The genes encoding the enzymes of the pentosophosphate pathway (1–7) in *T. bogorovii* BBS.

№	Function Number, Enzyme Annotation, and Number (According to KEGG Orthology Database)	Gene ID	Gene Name and Annotation Presented in GenBank
1	K00845 glucokinase [EC:2.7.1.2]	LT988_04670	*glk*; glucokinase
2	K01810 glucose-6-phosphate isomerase [EC:5.3.1.9]	LT988_04445	*pgi*; glucose-6-phosphate isomerase
2	K01810 glucose-6-phosphate isomerase [EC:5.3.1.9]	LT988_25180	*pgi*; glucose-6-phosphate isomerase
3	K21071 ATP-dependent phosphofructokinase/diphosphate-dependent phosphofructokinase [EC:2.7.1.11; 2.7.1.90]	LT988_14085	6-phosphofructokinase
4	K01623 fructose-bisphosphate aldolase, class I [EC:4.1.2.13]	LT988_25065	fructose-bisphosphate aldolase class I
4	K01624 fructose-bisphosphate aldolase, class II [EC:4.1.2.13]	LT988_08070	*fba*; fructose-bisphosphate aldolase class II
5	K01803 triose-phosphate isomerase (TIM) [EC:5.3.1.1]	LT988_02660	*tpiA*; triose-phosphate isomerase
6	K00134 glyceraldehyde 3-phosphate dehydrogenase (phosphorylating) [EC:1.2.1.12]	LT988_08050	*gap*; type I glyceraldehyde-3-phosphate dehydrogenase
7	K00927 phosphoglycerate kinase [EC:2.7.2.3]	LT988_08060	phosphoglycerate kinase
8	K15634 2,3-bisphosphoglycerate-dependent phosphoglycerate mutase [EC:5.4.2.11]	LT988_13795	histidine phosphatase family protein
8	K15633 2,3-bisphosphoglycerate-independent phosphoglycerate mutase [EC:5.4.2.12]	LT988_06575	*gpmI*; 2,3-bisphosphoglycerate-independent phosphoglycerate mutase
9	K01689 enolase [EC:4.2.1.11]	LT988_19115	*eno*; phosphopyruvate hydratase
10	K00873 pyruvate kinase [EC:2.7.1.40]	LT988_01025	*pyk*; pyruvate kinase
10	K00873 pyruvate kinase [EC:2.7.1.40]	LT988_08065	*pyk*; pyruvate kinase
10	K00873 pyruvate kinase [EC:2.7.1.40]	LT988_22860	pyruvate kinase
11	K03841 fructose-1,6-bisphosphatase I [EC:3.1.3.11]	LT988_07425	class 1 fructose-bisphosphatase
12	K00172 pyruvate-ferredoxin oxidoreductase gamma subunit [EC:1.2.7.1]	LT988_13550	2-oxoacid:acceptor oxidoreductase family protein
	K00169 pyruvate-ferredoxin oxidoreductase alpha subunit [EC:1.2.7.1]	LT988_13555	hypothetical protein
	K00170 pyruvate-ferredoxin oxidoreductase beta subunit [EC:1.2.7.1]	LT988_13560	thiamine pyrophosphate-dependent enzyme
12	K03737 pyruvate-ferredoxin/flavodoxin oxidoreductase [EC:1.2.7.1 1.2.7.-]	LT988_24065	2-oxoacid:acceptor oxidoreductase family protein
	K03737 pyruvate-ferredoxin/flavodoxin oxidoreductase [EC:1.2.7.1 1.2.7.-]	LT988_24180	*nifJ*; pyruvate:ferredoxin (flavodoxin) oxidoreductase
13	K00163 pyruvate dehydrogenase E1 component [EC:1.2.4.1]	LT988_22730	*aceE*; pyruvate dehydrogenase (acetyl-transferring), homodimeric type
14	K00382 dihydrolipoamide dehydrogenase [EC:1.8.1.4]	LT988_05285	dihydrolipoyl dehydrogenase
14	K00382 dihydrolipoamide dehydrogenase [EC:1.8.1.4]	LT988_13640	dihydrolipoyl dehydrogenase
14	K00382 dihydrolipoamide dehydrogenase [EC:1.8.1.4]	LT988_22720	*lpdA*; dihydrolipoyl dehydrogenase
15	K00627 pyruvate dehydrogenase E2 component (dihydrolipoamide acetyltransferase) [EC:2.3.1.12]	LT988_22725	*aceF*; dihydrolipoyllysine-residue acetyltransferase

**Table 8:** The genes encoding the enzymes involved in Sox system thiosulfate oxidation (1–5), sulfide oxidation (sulfide/quinone oxidoreductase (SQR (6)), and flavocytochrome *c* sulfide dehydrogenase (FccAB (7,8)) in *T. bogorovii* BBS.

№	Function Number, Enzyme Annotation, and Number (According to KEGG Orthology Database)	Gene ID	Gene Name and Annotation Presented in GenBank
1	K17222 L-cysteine S-thiosulfotransferase [EC:2.8.5.2]	LT988_12625	*soxA*; sulfur oxidation c-type cytochrome SoxA
1	K17222 L-cysteine S-thiosulfotransferase [EC:2.8.5.2]	LT988_24245	*soxA*; sulfur oxidation c-type cytochrome SoxA
2	K17223 L-cysteine S-thiosulfotransferase [EC:2.8.5.2]	LT988_12630	*soxX*; sulfur oxidation c-type cytochrome SoxX
2	K17223 L-cysteine S-thiosulfotransferase [EC:2.8.5.2]	LT988_24240	*soxX*; sulfur oxidation c-type cytochrome SoxX
3	K17224 S-sulfosulfanyl-L-cysteine sulfohydrolase [EC:3.1.6.20]	LT988_24235	*soxB*; thiosulfohydrolase SoxB
4	K17226 sulfur-oxidizing protein SoxY	LT988_05335	*SoxY*; thiosulfate oxidation carrier protein SoxY
4	K17226 sulfur-oxidizing protein SoxY	LT988_16035	*SoxY*; thiosulfate oxidation carrier protein SoxY
4	K17226 sulfur-oxidizing protein SoxY	LT988_21280	hypothetical protein
5	K17227 sulfur-oxidizing protein SoxZ	LT988_05330	*soxZ*; thiosulfate oxidation carrier complex protein SoxZ
5	K17227 sulfur-oxidizing protein SoxZ	LT988_16040	*soxZ*; thiosulfate oxidation carrier complex protein SoxZ
5	K17227 sulfur-oxidizing protein SoxZ	LT988_21285	*soxZ*; thiosulfate oxidation carrier complex protein SoxZ
6	K17218 sulfide:quinone oxidoreductase [EC:1.8.5.4]	LT988_02950	FAD-dependent oxidoreductase
6	K17218 sulfide:quinone oxidoreductase [EC:1.8.5.4]	LT988_15980	FAD-dependent oxidoreductase
7	K17230 cytochrome subunit of sulfide dehydrogenase	LT988_00620	cytochrome *c*4
8	K17229 sulfide dehydrogenase [flavocytochrome *c*] flavoprotein chain [EC:1.8.2.3]	LT988_00625	FCSD flavin-binding domain-containing protein

**Table 9:** Genes encoding Dsr proteins of *T. bogorovii* BBS and *Alc. vinosum* DSM 180^T^.

№	ID and Name of *Alc. vinosum* DSM 180T Gene	Function Number, Enzyme Annotation, and Number of *T. bogorovii* BBS (According to KEGG Orthology Database)	Gene ID of *T. bogorovii* BBS	Gene name and Annotation of *T. bogorovii* BBS Presented in GenBank
1	Alvin_1265 *dsrS*	no KO assigned	LT988_06595	sulfur reduction protein DsrS
2	Alvin_1264 *dsrR*	K13628 iron–sulfur cluster assembly protein	LT988_06600	iron–sulfur cluster assembly accessory protein
3	Alvin_1263 *dsrN*	K02224 cobyrinic acid a,c-diamide synthase [EC:6.3.5.9 6.3.5.11]	LT988_06605	cobyrinate a,c-diamide synthase
4	Alvin_1262 *dsrP*	no KO assigned	LT988_06610	*nrfD*; polysulfide reductase NrfD
5	Alvin_1261 *dsrO*	no KO assigned	LT988_06615	4Fe-4S dicluster domain-containing protein
6	Alvin_1260 *dsrJ*	no KO assigned	LT988_06620	sulfur reduction protein DsrJ
7	Alvin_1259 *dsrL*	no KO assigned	LT988_06625	NAD(P)-binding protein
8	Alvin_1258 *dsrK*	no KO assigned	LT988_06630	(Fe-S)-binding protein
9	Alvin_1257 *dsrM*	K00374 nitrate reductase gamma subunit [EC:1.7.5.1 1.7.99.-]	LT988_06635	respiratory nitrate reductase subunit gamma
10	Alvin_1256 *dsrC*	K23077 dissimilatory sulfite reductase related protein	LT988_06640	TusE/DsrC/DsvC family sulfur relay protein
11	Alvin_1255 *dsrH*	K07237 tRNA 2-thiouridine synthesizing protein B	LT988_06645	*tusB*; sulfurtransferase complex subunit TusB
12	Alvin_1254 *dsrF*	K07236 tRNA 2-thiouridine synthesizing protein C	LT988_06650	*tusC*; sulfurtransferase complex subunit TusC
13	Alvin_1253 *dsrE*	K07235 tRNA 2-thiouridine synthesizing protein D [EC:2.8.1.-]	LT988_06655	*tusD*; sulfurtransferase complex subunit TusD
14	Alvin_1252 *dsrB*	K11181 dissimilatory sulfite reductase beta subunit [EC:1.8.99.5]	LT988_06660	*dsrB*; dissimilatory-type sulfite reductase subunit beta
15	Alvin_1251 *dsrA*	K11180 dissimilatory sulfite reductase alpha subunit [EC:1.8.99.5]	LT988_06665	*dsrA*; dissimilatory-type sulfite reductase subunit alpha

## Data Availability

Data are contained within the article.

## Abbreviations

Aa, aa—amino acids
*Alc. vinosum*—*Allochromatium vinosum*
ANI—Average Nucleotide Identity
ATCC—American Type Culture Collection
ATP—adenosine triphosphate
bp—base pairs
CRISPR—clustered regularly interspaced short palindromic repeats
DDH—digital DNA-DNA hybridization parameter
DNA—deoxyribonucleic acid
e-value—the expectation value
FAD—flavin adenine dinucleotide
FccAB—flavocytochrome c sulfide dehydrogenase
FCSD—flavocytochrome c sulfide dehydrogenase
GC content—guanine-cytosine content
HiPIP—High potential iron–sulfur protein
kbp—kilobase pairs
kDa—kilodalton
KEGG—Kyoto Encyclopedia of Genes and Genomes
LH—light-harvesting antenna complex
LPSN—List of Prokaryotic names with Standing in Nomenclature
Mb—megabase
Mo-containing—molybdenum-containing
NAD—nicotinamide adenine dinucleotide
NAD+—the oxidized form
NADH—the reduced form
NADP—nicotinamide adenine dinucleotide phosphate
NAD(P)H—the reduced form
ncRNAs—non-coding RNA
ONT—Oxford Nanopore Technologies
PEP—phosphoenolpyruvate
PRK—Phosphoribulokinase
PTS—phosphotransferase system
PufX—intrinsic membrane protein
RC—reaction center
Rhd—rhodanese-like protein
RLP—RubisCO-like protein
RNA—ribonucleic acid
rRNA—ribosomal RNA
rTCA—reductive tricarboxylic acid cycle
RuBisCO—Ribulose-1,5-bisphosphate carboxylase/oxygenase
SBP—Sedoheptulose-bisphosphatase
Sgp—sulfur globule proteins
Sox system—sulfur oxidation system
SQR—sulfide/quinone oxidoreductase
*T. bogorovii*—*Thiocapsa bogorovii*
*T. imhoffii*—*Thiocapsa imhoffii*
*T. marina*—*Thiocapsa marina*
*T. rosea*—*Thiocapsa rosea*
*T. roseopersicina*—*Thiocapsa roseopersicina*
TCA cycle—the tricarboxylic acid cycle
tRNA—transfer RNA
TsdA—tetrathionate reductase
